# Surgical Experiences in the Present War

**Published:** 1914-12-19

**Authors:** 


### SURGICAL EXPERIENCES IN THE PRESENT WAR.

### Modern Bullet Wounds of the Soft Parts.

THE surgical history of the war will be a very elaborate compilation and will contain chapters both numerous and varied. The scale on which wounds have been inflicted—and the experience is by no means concluded—is, of course, an enormous one. The forms of injury also are very numerous. Apart from wounds caused by the bayonet, lance, sword, and rifle-bullet, there are the consequences of the great variety of missiles used by modern artillery. And, again, it may be fairly claimed that never before has there been a war in which there have been so many highly competent surgical experts ready to make exact observations and to form sound conclusions. It may be said, further, that the organisation of the hospital services is at a level which permits the pursuit of these highly desirable ends under not unfavourable conditions. In short, looking at the war as a great field for the cultivation of surgical truth, the opportunities are unprecedented and the numbers of students are great beyond parallel. It is impossible, therefore, to doubt that when the complete story comes to be compiled, many great and important lessons will emerge. The final compilation is no doubt far distant, but the contributions out of which it will be shaped are gradually accumulating, and though these must yet be tested by further experience, some of them are already taking a shape which has at least the promise of finality.

Amongst experiences which claim special attention as resting on authority of the highest order is a note on "Wounds of the Soft Parts produced by the Modern Bullet," by Colonel G. H. Makins, C.B., and published in the *Lancet* of the 12th inst. It is not necessary to remind our readers of the peculiar worth which a contribution from such a source must certainly possess. When a highly experienced surgeon having previous knowledge of war on a large scale tells us what he has observed in discharging great responsibilities in the present war, everyone will recognise that the contribution is one of the testimonies vital to the occasion. It is first-hand knowledge gained amidst special opportunities by an observer of specially trained capacity and judgment. Colonel Makins' paper, in other words, is one of the authoritative documents out of which, in due course, the complete surgical history of the war will be constructed. In some of its aspects it has an appeal to the general as well as to the professional reader, and these may here be presented.

In connection with bullet wounds inflicted during the war there has been more or less newspaper accusation, both on one side and the other, of the use of bullets of the "expanding" or "soft-nosed" variety. The accusation is based generally on the extent of the damage inflicted by a bullet on the soft tissues, and particularly perhaps on the large size and ragged character of the wound of exit. The question, in addition to its surgical interest, is of international importance, seeing that the use of this type of bullet is forbidden by the laws of civilised warfare. In this respect it is essential to note what Colonel Makins has written. His statement, in brief, is that in certain circumstances the modern pointed bullet may wound the soft parts more severely than has ever been noted in the case of expanding or soft-nosed bullets. With a small ordinary-looking aperture of entry, the exit wound may be very large, may be oval or stellate in shape, and may show a protruding mass of torn muscles and other soft tissues; all these characters, be it noted, being possible without any evidence that part of the damage is due to fragments of bone produced by fracture or splintering caused by the bullet. It is therefore easy to understand that an untrained observer, face to face with such injuries, may honestly come to the conclusion that they undoubtedly mean the use by the enemy of explosive or "dum-dum" bullets.

Now what is the explanation of the fact that the modern pointed bullet may simulate in its effects those believed to be characteristic of bullets of the soft-nosed variety? It is found in the tendency of the former, under certain conditions, to revolve around its transverse axis. When such a bullet strikes the surface of the body fairly, as one may say—that is, at right angles to the surface—it produces a small, clean entrance wound and a small, clean exit wound, with a narrow bore or canal connecting these. But if the bullet strikes the surface at an acute angle, the clean penetrating force of its sharp tip is to some extent lost. The resistance offered to the slope of the sharp end of the bullet is not equal at all points of its circumference, and in these circumstances the bullet is apt to revolve round its transverse axis. Hence, while it may make quite a small entrance aperture, its progress may be not "end on," so to speak, but may be accomplished in such a fashion that its relatively large lateral surface is driven forcibly against and through the soft parts. In this fashion it may come about that the sharp-pointed bullet may, with a small entrance wound, lead to considerable damage to the deeper tissues and to a large and more or less irregular aperture of exit.

There are certain facts of observation which go to show the ease with which the sharp-pointed bullet is deflected, and which support, therefore, the suggestion that such a bullet, when it strikes the surface obliquely, may, from rotation round its transverse axis, cause damage suggestive of the use of the explosive variety. One of these facts is that occasionally an entrance wound having the shape of the lateral aspect of the bullet is seen. A second is that retained bullets are sometimes found lying with the long axis at right angles to the track of the wound; or the bullet may have made a complete half-turn and so have its base directed towards the entrance aperture. And thirdly, in a large proportion of head injuries it is noted that the bullet has not penetrated the skull, but has traced a "gutter" on its surface—that is, even though travelling with considerable velocity, it has been readily deflected from its original course. These experiences, we repeat, are of general as well as of surgical interest, and we think it right they should be widely known. Charges of using "dum-dum" bullets have been brought against the enemy by all the combatants. If they can be supported by adequate evidence the time will come when the appropriate authority will pronounce on them. But it is obvious that conclusions based on the character of wounds, in so far as these rest on amateur or untrained observations, have but an insecure basis. In this relation it may be recalled that Colonel Makins, in addition to his general experience, has special knowledge of the effects of soft-nosed bullets, as he had the opportunity of studying these in the South African campaign. There is one other point on which we may add a word. In a recent message from the "Eye-Witness" of our Expeditionary Force we were told that trench warfare now includes the use of hand grenades, which were described as "murderous missiles," in spite of certain amusing incidents which accompanied their use. A certain number of men wounded in this fashion have now come under observation, and it is so far satisfactory to read Colonel Makins' conclusion that the "hand grenade" appears to cause more damage to the trench than to the occupants of the trench. On our side, at all events, we trust this experience may be continued.

